# Supplementary material for: Influence of the COVID-19 pandemic on running behaviors, motives, and running-related injury: A one-year follow-up survey
Source: PLoS One. 2022 Mar 30;17(3):e0264361. doi: 10.1371/journal.pone.0264361 (PMC8967014; doi:10.1371/journal.pone.0264361)
Supplement: S1 File — (DOCX) [file pone.0264361.s001.docx]

Supplementary File. Follow-Up COVID-19 Running Survey

Start of Block: Follow-Up Survey

Q52 You are receiving this follow-up survey as you previously signed a consent form to complete a study assessing the effects of the COVID-19 pandemic on running behaviors and running-related injury, and indicated you would be willing to complete a follow-up survey. This follow-up survey fall under the same UVA IRB-SBS Protocol # 3677. By completing this follow-up survey, your consent is implied given your original consent to participate. 
 
This follow-up survey will take about 10-15 minutes of your time. Your participation in the study is completely voluntary.  You may stop completing the survey at any time by closing out of the browser. There is no penalty for withdrawing. 
 
**Thank you for your willingness to participate in this follow-up survey! If you have questions about the study, contact:**
Alexandra DeJong (Study Coordinator)
Department of Kinesiology, 210 Emmet Street S
University of Virginia, Charlottesville, VA 22903.  
Telephone: (434) 924-6184
afd4au@virginia.eduDr. Jay Hertel (PI)
Department of Kinesiology, 210 Emmet Street S
University of Virginia, Charlottesville, VA 22903.  
Telephone: (434) 243-8673
jnh7g@virginia.edu**To obtain more information about the study, ask questions about the research procedures, express concerns about your participation, or report illness, injury or other problems, please contact:**
Tonya R. Moon, Ph.D.
Chair, Institutional Review Board for the Social and Behavioral Sciences
One Morton Dr Suite 500 
University of Virginia, P.O. Box 800392
Charlottesville, VA 22908-0392
Telephone:  (434) 924-5999 
Email: irbsbshelp@virginia.edu
Website: https://research.virginia.edu/irb-sbs
Website for Research Participants: https://research.virginia.edu/research-participants

Q3 Please provide your full email address below ***(please provide the same email address as you received the survey email from so we are able to match responses from last year)***:

________________________________________________________________

End of Block: Follow-Up Survey

Start of Block: Running behaviors in the past 12 months

Q4 Please respond to the next set of questions in regards to your running behaviors ***in the past 12 months*during the COVID-19 pandemic.**

| Page Break |  |
| --- | --- |

Q5 **In the past 12 months during the COVID-19 pandemic,** how many times per week did you perform:

|  | 0 | 1 | 2 | 3 | 4 | 5 | 6 | 7 | 8 | 9 | 10 | 11 | 12 | 13 | 14 |
| --- | --- | --- | --- | --- | --- | --- | --- | --- | --- | --- | --- | --- | --- | --- | --- |

| All Runs () | 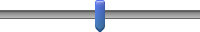 |
| --- | --- |
| Sustained Runs () | 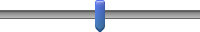 |
| Workouts (I.e. track intervals, fartleks) () | 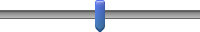 |
| Cross-Training Activities (I.e. walking, yoga, strength training, cycling, swimming) () | 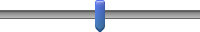 |

Q6 **In the past 12 months during the COVID-19 pandemic,**what was your typical running pace (minutes/miles) for:

|  |  |
| --- | --- |
| Sustained Runs (77) | ▼ N/A (88) ... 14:00 (86) |
| Workouts (ie. fartleks, intervals, speed training) (78) | ▼ N/A (88) ... 14:00 (86) |

Q7 How many miles per week on average did you run**after social distancing restrictions were lifted in your region following the COVID-19 pandemic?**

▼ 0 (1) ... greater than 120 (104)

Q8 Was your average weekly mileage different than anticipated over the past 12 months, in comparison to previous years of running training?

- Yes (1)
- No (2)
- Not applicable to me (3)

Q9 **After social distancing restrictions were lifted in your region following the COVID-19 pandemic,** what were your motives for running (select all that apply):

- Exercise/Fitness (1)
- Competition/Races (2)
- Socialization (3)
- Stress Relief (4)
- Enjoyment/Pleasure (5)
- Occupy Free Time (6)

Q10 **After social distancing restrictions were lifted in your region following the COVID-19 pandemic,** did you primarily perform runs indoors, outdoors, or both?

- Indoors (1)
- Outdoors (2)
- Both (4)

Q11 **In the past 12 months during the COVID-19 pandemic,** did you at any point lose access to your typical training environment (i.e. access to preferred trails, paths, tracks, gyms)?

- Yes (1)
- No (2)

Display This Question:

If In the past 12 months during the COVID-19 pandemic, did you at any point lose access to your typi... = Yes

Q24 **In the past 12 months during the COVID-19 pandemic,** were you able to return at any point to running on preferred ***trails***?

- Yes (1)
- No (2)
- N/A I do not run on trails (3)
- N/A I did not lose access to my preferred trails (4)

Display This Question:

If In the past 12 months during the COVID-19 pandemic, did you at any point lose access to your typi... = Yes

Q25 **In the past 12 months during the COVID-19 pandemic,** were you able to return at any point to running on preferred ***sidewalk or road paths***?

- Yes (1)
- No (2)
- N/A I do not run on sidewalk or road paths (3)
- N/A I never lost access to my preferred sidewalk or road paths (4)

Display This Question:

If In the past 12 months during the COVID-19 pandemic, did you at any point lose access to your typi... = Yes

Q26 **In the past 12 months during the COVID-19 pandemic,** were you able to return at any point to running on preferred ***tracks***?

- Yes (1)
- No (2)
- N/A I do not run on tracks (3)
- N/A I never lost access to a track (5)

Display This Question:

If In the past 12 months during the COVID-19 pandemic, did you at any point lose access to your typi... = Yes

Q27 **In the past 12 months during the COVID-19 pandemic,** were you able to return at any point to running in preferred ***gyms***?

- Yes (1)
- No (2)
- N/A I do not run in a gym (3)
- N/A I never lost access to a gym (4)

Q12 **In the past 12 months during the COVID-19 pandemic,**did you primarily perform runs alone, in groups, or both?

- Alone (1)
- Groups (2)
- Both (4)

Display This Question:

If In the past 12 months during the COVID-19 pandemic, did you primarily perform runs alone, in grou... = Alone

Q28 **In the past 12 months during the COVID-19 pandemic,**were you able to return at any point to running in a group?

- Yes (1)
- No (2)
- N/A I never run in groups (3)

Q13 **In the past 12 months during the COVID-19 pandemic,**did you use any form of technology to track your runs (Ie. smart watch, running phone application)?

- Yes (1)
- No (2)

Q14 **In the past 12 months during the COVID-19 pandemic,**what time of day did you typically run? (select all that apply)

- Early morning (5am-7am) (1)
- Mid-Morning (8am-10am) (2)
- Midday (11am-1pm) (3)
- Early Afternoon (2pm-4pm) (4)
- Afternoon (5pm-7pm) (5)
- Evening (8pm-10pm) (6)
- Night Runs (11pm-4am) (7)

Q15 How much would you say your running training has changed **in the past 12 months during the COVID-19 pandemic?**

- It has increased a great deal (1)
- It has increased a lot (2)
- It has increased a moderate amount (3)
- It has increased a little (4)
- No change at all (5)
- It has decreased a little (6)
- It has decreased a moderate amount (7)
- It has decreased a lot (8)
- It has decreased a great deal (9)

Q16 How concerned are you about how the COVID-19 pandemic has affected your ***running training*** **from the past 12 months?**

- Very concerned (1)
- Somewhat concerned (2)
- Neutral (3)
- Somewhat unconcerned (4)
- Very unconcerned (5)

Q17 How concerned are you about how the COVID-19 pandemic has affected your ***running goals* from the past 12 months**?

- Very concerned (1)
- Somewhat concerned (2)
- Neutral (3)
- Somewhat unconcerned (4)
- Very unconcerned (5)

Q45 In the past 12 months during the COVID-19 pandemic, did you participate in any virtual races?

- Yes (1)
- No (2)

Display This Question:

If In the past 12 months during the COVID-19 pandemic, did you participate in any virtual races? = Yes

Q46 How many virtual races did you complete?

▼ 1 (1) ... More than 20 (21)

Display This Question:

If In the past 12 months during the COVID-19 pandemic, did you participate in any virtual races? = Yes

Q47 What distance(s) were the virtual races that you ran? Select all the apply:

- 5-K (3.1 miles) (1)
- 8-K (4.9 miles) (2)
- 10-K (6.2 miles) (3)
- 15-K (9.3 miles) (4)
- 16-K (10 miles) (5)
- 20-K (12 miles) (6)
- 21.1-K (half marathon) (7)
- 30-K (19 miles) (8)
- 42.2-K (marathon) (9)
- 50-K (31 miles) (10)
- over 50-K race (ultra-marathon distances) (11)
- Other: (13) ________________________________________________

Q48 **In the past 12 months during the COVID-19 pandemic,** did you participate in any in-person races?

- Yes (1)
- No (2)

Display This Question:

If In the past 12 months during the COVID-19 pandemic, did you participate in any in-person races? = Yes

Q49 How many in-person races did you complete?

▼ 1 (1) ... More than 20 in-person races (21)

Display This Question:

If In the past 12 months during the COVID-19 pandemic, did you participate in any in-person races? = Yes

Q50 What distance(s) were the in-person races that you ran? Select all the apply:

- 5-K (3.1 miles) (1)
- 8-K (4.9 miles) (2)
- 10-K (6.2 miles) (3)
- 15-K (9.3 miles) (4)
- 16-K (10 miles) (5)
- 20-K (12 miles) (6)
- 21.1-K (half marathon) (7)
- 30-K (19 miles) (8)
- 42.2-K (marathon) (9)
- 50-K (31 miles) (10)
- over 50-K race (ultra-marathon distances) (11)
- Other: (12) ________________________________________________

End of Block: Running behaviors in the past 12 months

Start of Block: Injury Status During COVID-19

Q18 **In the past 12 months during the COVID-19 pandemic,**did you suffer from any running-related injuries?

- Yes (1)
- No (2)

Skip To: End of Block If In the past 12 months during the COVID-19 pandemic, did you suffer from any running-related injur... = No

Q19 How many running-related injuries did you sustain**in the past 12 months during the COVID-19 pandemic?**

▼ 1 (1) ... Greater than 10 (11)

Q20 How long were you unable to perform running training due to running-related injuries sustained **in the past 12 months during the COVID-19 pandemic?**

▼ 1 day (1) ... 1-2 months (22)

Q23 How long did you have to modify your running training due to running-related injuries sustained **in the past 12 months during the COVID-19 pandemic?**

▼ 1 day (1) ... 11-12 months (16)

Q21 For the running-related injuries you sustained **in the past 12 months during the COVID-19 pandemic,**please type the number of injury types that occurred at each body location in the text boxes provided.

|  | Injury Type | | | |
| --- | --- | --- | --- | --- |
|  | Sprain (ligament) (1) | Strain (muscle or tendon) (2) | Fracture (broken bone) (3) | Other (please explain) (4) |
| Toe (1) |  |  |  |  |
| Foot (10) |  |  |  |  |
| Ankle (2) |  |  |  |  |
| Lower leg (3) |  |  |  |  |
| Knee (4) |  |  |  |  |
| Thigh (5) |  |  |  |  |
| Hamstring (6) |  |  |  |  |
| Hip (7) |  |  |  |  |
| Groin (11) |  |  |  |  |
| Abdomen (8) |  |  |  |  |
| Low Back (9) |  |  |  |  |

End of Block: Injury Status During COVID-19

Start of Block: Running behaviors in the past month

Q31 Please respond to the next set of questions ***in regards to your running behaviors in the last month.***

| Page Break |  |
| --- | --- |

Q32 **In the past month,** how many times per week did you perform:

|  | 0 | 1 | 2 | 3 | 4 | 5 | 6 | 7 | 8 | 9 | 10 | 11 | 12 | 13 | 14 |
| --- | --- | --- | --- | --- | --- | --- | --- | --- | --- | --- | --- | --- | --- | --- | --- |

| All Runs () | 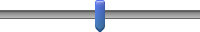 |
| --- | --- |
| Sustained Runs () | 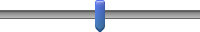 |
| Workouts (I.e. track intervals, fartleks) () | 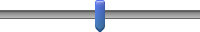 |
| Cross-Training Activities (I.e. walking, yoga, strength training, cycling, swimming) () | 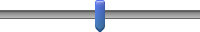 |

Q33 **In the past month,** what was your typical running pace (minutes/miles) for:

|  |  |
| --- | --- |
| Sustained Runs (77) | ▼ N/A (88) ... 14:00 (86) |
| Workouts (ie. fartleks, intervals, speed training) (78) | ▼ N/A (88) ... 14:00 (86) |

Q34 How many miles per week on average did you run**in the past month?**

▼ 1 (1) ... 100+ (100)

Q35 Has your mileage been different than expected around this current timeframe in your training plan?

- Yes (1)
- No (2)
- Not applicable to me (3)

Q36 **In the past month,** what were your motives for running (select all that apply):

- Exercise/Fitness (1)
- Competition/Races (2)
- Socialization (3)
- Stress Relief (4)
- Enjoyment/Pleasure (5)
- Occupy Free Time (6)

Q37 **In the past month,** did you primarily perform runs indoors, outdoors, or both?

- Indoors (1)
- Outdoors (2)
- Both (4)

Q41 **In the past month,** what time of day did you typically run? (select all that apply)

- Early morning (5am-7am) (1)
- Mid-Morning (8am-10am) (2)
- Midday (11am-1pm) (3)
- Early Afternoon (2pm-4pm) (4)
- Afternoon (5pm-7pm) (5)
- Evening (8pm-10pm) (6)
- Night Runs (11pm-4am) (7)

Q42 How much would you say your running training has changed **in the past month?**

- It has increased a great deal (1)
- It has increased a lot (2)
- It has increased a moderate amount (3)
- It has increased a little (4)
- No change at all (5)
- It has decreased a little (6)
- It has decreased a moderate amount (7)
- It has decreased a lot (8)
- It has decreased a great deal (9)

Q43 How concerned are you about how the COVID-19 pandemic is **currently** affecting your ***running training***?

- Very concerned (1)
- Somewhat concerned (2)
- Neutral (3)
- Somewhat unconcerned (4)
- Very unconcerned (5)

Q44 How concerned are you about how the COVID-19 pandemic is **currently** affecting your ***running goals***?

- Very concerned (1)
- Somewhat concerned (2)
- Neutral (3)
- Somewhat unconcerned (4)
- Very unconcerned (5)

Q51 Please feel free to provide additional comments about how COVID-19 affected your running training, behaviors, and/or running health in the past year:

________________________________________________________________

________________________________________________________________

________________________________________________________________

________________________________________________________________

________________________________________________________________

End of Block: Running behaviors in the past month
